# DNA methylation profile is associated with the osteogenic potential of three distinct human odontogenic stem cells

**DOI:** 10.1038/s41392-017-0001-6

**Published:** 2018-01-12

**Authors:** Tingting Ai, Jieni Zhang, Xuedong Wang, Xiaowen Zheng, Xueyan Qin, Qian Zhang, Weiran Li, Wei Hu, Jiuxiang Lin, Feng Chen

**Affiliations:** 1 Department of Orthodontics, School of Stomatology, Peking University, Beijing, 100081 China; 2 Central Laboratory, School of Stomatology, Peking University, Beijing, 100081 China; 3 Present Address: Department of Orthodontics, Guanghua School and Hospital of Stomatology & Institute of Stomatological Research, Sun Yat-sen University, Guangzhou, China

## Abstract

Among the various sources of human autologous stem cells, stem cells isolated from dental tissues exhibit excellent properties in tissue engineering and regenerative medicine. However, the distinct potential of these odontogenic cell lines remains unclear. In this study, we analyzed DNA methylation patterns to determine whether specific differences existed among three different odontogenic cell types. Using the HumanMethylation450 Beadchip, the whole genomes of human dental pulp stem cells (DPSCs), periodontal ligament stem cells (PDLSCs), and dental follicle progenitor cells (DFPCs) were compared. Then, the osteogenic potential of these cells was evaluated both in vitro and in vivo, and the methylation levels of certain genes related to bone formation differed among the three cell lines. *P* values less than 0.05 were considered to indicate statistical significance. The three cell types showed highly similar DNA methylation patterns, although specific differences were identified. Gene ontology analysis revealed that one of the most significantly different gene categories was related to bone formation. Thus, expression of cell surface epitopes and osteogenic-related transcription factors as well as the bone formation capacity were compared. The results showed that compared with DFPCs and DPSCs, PDLSCs had higher transcription levels of osteogenic-related factors, a higher in vitro osteogenic potential, and an increased new bone formation capacity in vivo. In conclusion, the results of this study suggested that the differential DNA methylation profiles could be related to the osteogenic potential of these human odontogenic cell populations. Additionally, the increased osteogenic potential of PDLSCs might aid researchers or clinicians in making better choices regarding tissue regeneration and clinical therapies.

## Stem cells: Differences in bone-forming potential of dental stem cells

Stem cells isolated from the periodontal ligament are better at making new bone tissue than other kinds of dental stem cells. Feng Chen and colleagues from Peking University, China, analyzed the profile of DNA methylation that affect gene expression in stem cells isolated from human dental pulp (found in the center of the tooth) or periodontal ligaments (which attach tooth roots to bone), as well as from dental follicle progenitor cells that eventually form periodontal ligament cells. They saw significant differences in DNA methylation among genes related to bone production. They thus tested the bone-forming ability of each cell type, both in cell culture experiments and mice. The periodontal ligament stem cells proved the best, suggesting they could be useful for tissue engineering and regenerative medicine.

## Introduction

Due to advances in biological tissue engineering, the use of autologous mesenchymal stem/stromal cells (MSCs) as biological material seed has become increasingly popular. MSCs have recently been a particular focus in many areas of scientific research^1^. These stem cells are believed to be excellent candidates for tissue engineering approaches and represent the future of clinical stem-cell-based bone regeneration^2^. In addition, the developments in the field have yielded promising prospects for the use of MSCs in clinical trials^3^. The various sources of autologous stem cells include a specific postnatal stem cell line isolated from dental tissue, embryonic and adult stem cells derived from bone marrow, umbilical cord blood, and amniotic fluid^4^. Tooth-derived stem cell populations comprise a high percentage of progenitor cells and an excellent bone regeneration capacity. Thus, many studies have focused on odontogenic stem cells. To date, various human odontogenic stem/progenitor cell types have been isolated and identified, including dental pulp stem cells (DPSCs), periodontal ligament stem cells (PDLSCs), and dental follicle progenitor cells (DFPCs)^5^; all of which present MSC properties characterized by self-renewal, non-specialization, and multilineage differentiation potential; importantly, these cells display osteogenic capacity, which is one of the most crucial factors in tissue regeneration^6^. However, the osteogenic differentiation potential of these cells for bone regeneration may differ because these cells are derived from distinct tooth tissues and have different fates. Additionally, studies have investigated the optimal cell type for clinical use and/or basic research studies. For example, the differences between the proteomes of PDLSCs and DPSCs and the differences in other characteristics, such as the morphological properties, immune-phenotypes, or general gene expression patterns, among odontogenic stem cell lines^7–10^ have been reported. However, the conclusions of the above-mentioned studies were inconsistent. Thus, there is no basis for the application of various tooth-derived stem cell lines in tissue bioengineering.

Epigenetics is one of the most rapidly developing fields in the biological sciences^11^. The epigenetic regulation of tissue-specific gene expression includes DNA methylation and histone modifications, which are both highly crucial. The recent characterization of the human DNA methylome and CpG islands has accelerated the development of the field of epigenetics^12^. DNA methylation is essential for the regulation of gene expression profiles^13^. Early embryonic development and differentiation are regulated by and dependent on epigenetic markers, including DNA methylation.^14–16^ Additionally, DNA methylation provides a potential epigenetic mechanism for the preservation of the somatic progenitor state through repeated cell divisions^17^. Moreover, aberrant DNA methylation (hyper- or hypomethylation) could impact related gene expression and, thus, influence disease processes, such as cancer^18^. Thus, the alterations in DNA methylation patterns observed in certain conditions or diseases have increased the interest in the development of large-scale DNA methylation profiling methods and, hence, have facilitated technological breakthroughs^15^.

Therefore, the primary goal of this study was to identify the differences in the DNA methylation states among three dental stem cell lines. In addition, we aimed to determine whether the differential methylation profiles of these stem cells influence their potential.

## Methods

### Sample collection and cell culture

Three human-impacted third molars were used to obtain dental follicle tissue. Three premolars were extracted during orthodontic treatment and used to obtain dental pulp and periodontal ligaments. All subjects were generally systemically healthy. This study was approved by the Biomedical Ethics Committee of Peking University. Each subject signed an informed consent form before participating in the study. The mean age and gender distribution in each group were matched and did not significantly differ (Appendix Table 1).

To isolate and culture PDLSCs, the teeth were sterilized using fluoride and iodine and then rinsed with phosphate-buffered saline (PBS) three times. The PDL tissues were separated from the root surface. Then, the collected PDL tissues were minced and digested in a solution of 3 mg/mL collagenase I (Worthington Biochem, Freehold, NJ, USA) and 4 mg/mL dispase (Roche, Mannheim, Germany) for 1 h at 37 °C. PDLSCs from each donor were cultivated individually, and single-cell suspensions were obtained after centrifugation. The cell suspensions were then transferred to and seeded in 10-cm culture dishes (Costar, Cambridge, MA, USA) containing minimum essential medium (a-MEM) supplemented with 15% fetal bovine serum, 2 mmol/L glutamine, 100 U/mL penicillin, and 100 µg/mL streptomycin (all from Gibco BRL, Grand Island, NY, USA). The tissues were then incubated at 37 °C in a humidified atmosphere containing 5% CO_2_. Meanwhile, DFPCs from dental follicles were isolated and incubated using a procedure similar to that performed in PDLSCs. To isolate human DPSCs, dental pulp tissues were obtained from the dental pulp chamber and underwent subsequent digestion, centrifugation, and resuspension. Then, the cell suspension was seeded as described above.

### Characterization and morphological observation of DFPCs, PDLSCs, and DPSCs

After the isolation, suspension and seeding of the human DFPCs, PDLSCs, and DPSCs from the corresponding tissues of each donor, all three primary culture cell types were subjected to morphological identification and characterization. In our study, we mainly followed the detailed methods described in the Lancet paper published by Seo et al. for the identification and characterization of the three stem cell types. PDLSCs, DFPCs, and DPSCs were seeded separately in culture plates (BD, Franklin Lakes, NJ, USA) at a cell density of 5 × 10^4^ cells/mL and supplemented with a-MEM with 10% FBS. The images were captured under a phase-contrast inverted microscope (Nikon, Tokyo, Japan) after the cells had reached 80% confluency. The observed cell morphologies were similar to those previously reported and used to identify PDLSCs^19^, DPSCs, and DFPCs.

### DNA methylation assessment

The DNA methylation state in Passage 2 of the DPSCs, PDLSCs, and DFPCs was investigated using Infinium HumanMethylation450 (Infinium Methylation 450K; Illumina, Inc., CA, USA) to assess the methylation status of the whole genome. This 450K beadchip provides genome-wide coverage of 99% of the RefSeq genes characterized by more than 450,000 methylation sites, including both within and outside of CpG islands. Importantly, the coverage was targeted across genetic regions with sites in the promoter, 5′-untranslated region (UTR), first exon, gene body, and 3′-UTR, thereby providing a comprehensive view of the methylation status (Kobayashi et al.^15^). The genomic DNA was extracted using a QIAamp DNA mini kit (Qiagen, Hilden, Germany) accompanied by the recommended proteinase K and RNase A digestion. The genomic DNA (800 ng) was then treated with sodium bisulfite using a Zymo EZ DNA Methylation Kit™ (Zymo Research, Orange, CA, USA) according to the manufacturer’s instructions. An Illumina Infinium methylation assay was performed using 4 μL of bisulfite-converted genomic DNA at 50 ng/μL according to the Infinium HD methylation assay protocol. The data quality was confirmed using the GenomeStudio™ methylation module software (2010.3), and all samples conformed to the quality control. Uncorrected *b* values were extracted using the same software. The raw data were submitted to the Gene Expression Omnibus (GSE29290) database.

### Gene ontology (GO) analysis

After the methylation analysis, a gene ontology analysis was conducted to further classify the genes that significantly differentially expressed among the three Passage 2 cell groups. Novelbio software, which was obtained from the Novel Bioinformatics Co., Ltd. (Shanghai), was used for this analysis. This software integrates information from several databases, including NCBI, SwissProt/UniProt, PIR, IPI, GO, etc., marks the source and removes the repeated GO information. Then, a functional significance analysis was performed to classify the differential proteins, and the classification results were based on the significance of the discrete distribution analysis, misjudgment rate analysis, and enrichment analysis. Generally, Fisher’s exact test was performed to classify the GO categories, and the false discovery rate (FDR) was calculated to correct the *p* values. In addition, the enrichment analysis provided an evaluation of the significance of the functions as follows: as enrichment increased, the corresponding function was more specific. Within a significant category, the enrichment was expressed as follows: Re = (*n*_f_/*n*)/(*N*_f_/*N*), where *n*_f_ is the number of differential genes within a particular category, *n* is the total number of genes within the same category, *N*_f_ is the number of differential genes in the entire microarray, and *N* is the total number of genes in the microarray. Studying the distribution of proteins in GO can illustrate the functional representation of the differential proteins. Technical expertise regarding the systemic bioinformatic analysis was provided by Novel Bioinformatics Co., Ltd. (Shanghai).

### Flow cytometry

To compare the expression levels of the surface antigens, the cell suspensions were analyzed by flow cytometry. Briefly, ~5 × 10^5^ cells (Passage 4) were harvested, washed with PBS containing 1% bovine serum albumin (BSA), and incubated with antibodies against human STRO-1, CD146, and CD109 (BioLegend, San Diego, CA, USA) at 4 °C in the dark. The cell suspensions lacking the antibodies served as controls. After 45 min, the cells were washed twice with PBS containing 1% BSA. Finally, 500 μL of PBS supplemented with 1% BSA were added to the tubes, and the samples were subjected to a flow cytometric analysis using a BD Accuri™ C6 flow cytometer (BD Biosciences, San Jose, CA, USA).

### Real-time polymerase chain reaction (RT-PCR)

Total cellular RNA (Passage 2) was extracted from the cultured cells from each donor before differentiation using an RNeasy mini kit (QIAGEN GmbH, Hilden, Germany). Subsequently, cDNA was synthesized after a treatment with DNase I (Invitrogen, Carlsbad, CA). All reactions were performed simultaneously and included negative controls. The reaction mixtures were further incubated with RNase H at 37 °C for 20 min. Real-time PCR was performed using a SYBR Premix Ex Taq Kit (Takara) using a quantitative PCR System (CFX96, CA). GAPDH was used as a reference gene. In addition, the 2^−ΔΔCt^ method was used to evaluate and compare the expression levels (Livak and Schmittgen, 2001). The primers and corresponding probes used for the RT-PCR analysis were synthesized by the manufacturer (Sangon Biotech, China). The probes and primer sequences are listed in Appendix Table 2. The RT-PCR reactions were performed using the following parameters: 40 cycles: 94 °C for 3 min, 94 °C for 15 s, and 60 °C for 30 s.

### Alizarin Red S staining

All cell lines at Passage 4 were incubated in six-well plates at a density of 3 × 10^4^ per well and cultured with standard medium for 24 h, followed by osteo-inductive medium a-MEM supplemented with 10 nM dexamethasone, 50 μg/mL l-ascorbic acid-2-phosphate, 10 mM beta-glycerophosphate (Sigma-Aldrich, St. Louis, MO, USA), 10% FBS, and 1% antibiotic/antimycotic (Gibco BRL, Grand Island, NY, USA). The medium was refreshed at 2-day intervals. After 14 days of incubation, the cells were fixed with 70% ethanol for 15 min at room temperature and stained with Alizarin Red S at pH 4.2 (Sigma-Aldrich). After performing observations under an inverted phase-contrast microscope, the mineralized nodules were dissolved in hexadecylpyridinium chloride, and absorbance at 560 nm was quantified and statistically analyzed.

### Alkaline phosphatase activity

All cell groups at Passage 4 were incubated in six-well plates at a density of 3 × 10^4^ per well and cultured in standard medium for 24 h, followed by osteo-inductive medium refreshed at 2-day intervals. After 7 days, the cell samples were collected and disrupted ultrasonically to obtain protein, and the protein concentration was determined using a bicinchoninic acid protein assay (BCA reagent, Thermo Fisher Scientific, Inc., Waltham, MA, USA). The alkaline phosphatase (ALP) activity was detected using an ALP assay kit following the manufacturer’s instructions (Nanjing Jiancheng Bioengineering Institute, Nanjing, China).

### Transplantation

To investigate the in vivo osteogenic capacity of human DPSCs, PDLSCs, and DFPCs, subcutaneous transplantations of a cell mixture and hydroxyapatite were performed. The animal use was approved by the Animal Care Committee of Peking University Health Science Center. A mixture of ~5 × 10^6^ in vitro-expanded cells (Passage 4) and 1-cm^3^ hydroxyapatite was transplanted subcutaneously into the dorsal region of immune-deficient mice (Peking University Health Science Center, Animal Center, Beijing, China). The mice used were 6-week-old males (*n* = 3 per group; *n* = 1 for the BMSSC control). Each mouse had two transplantation sites. The amount of stem cells and hydroxylapatite for each cell type transplanted into the 6-week-old mice was identical to ensure that the external conditions were consistent. The animals were killed after 8 weeks, and the transplants were fixed in EDTA for over 24 h and then subjected to micro-CT scanning. In the scanned image, the white highlighted portion is the hydroxyapatite, and the gray–green highlighted portion is osteoid climbing around the hydroxyapatite. The darkest portion is soft tissue, which has a very low density and could be negligible. We enclosed the entire highlighted mass in a rectangle. In addition, the overall density of the hydroxyapatite plus newly formed osteoid was measured to represent the osteogenic ability of the stem cells.

### Statistical analysis

The in vitro experiments were performed in triplicate. In this study, the significance of the differences was determined using the Kruskal–Wallis test to analyze multiple groups. *P* values less than 0.05 were considered to indicate statistical significance.

## Results

### DNA methylation profiles significantly differed among DFPCs, PDLSC, and DPSCs

**Fig. 1:**
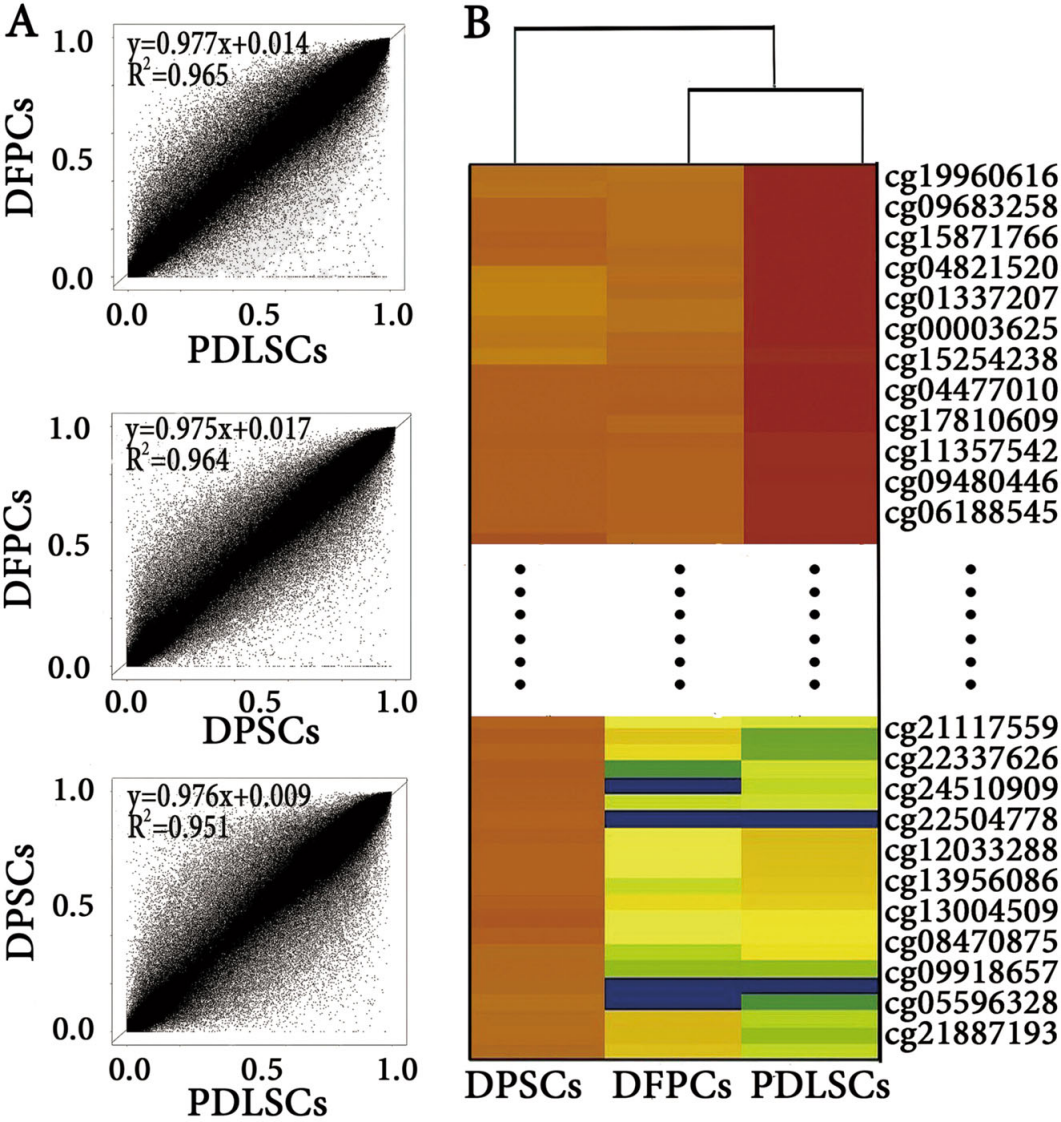
DNA methylation distribution in the genomes of DFPCs, PDLSCs, and DPSCs. **a** Scatter plots of CpG methylation *β* values between DFPCs and PDLSCs, DFPCs and DPSCs, and DPSCs and PDLSCs; *β* = *M*/(*M* + *U* + 100), where *M* and *U* represent the methylated and unmethylated signal intensities, respectively, and *R*^2^ is the relevant ratio for comparing two groups. The higher the ratio, the closer the relationship between the groups. **b** Cluster analysis of the most significantly different 12,054 CpG sites in DFPCs, PDLSCs, and DPSCs using a 450K DNA methylation microarray. The color label indicates that the different colors match different *β* values in the CpG sites in DFPCs, PDLSCs, and DPSCs. In addition, the colors between PDLSCs and DFPCs were closer than the colors between the other two pairs, indicating that the degree of the gene enrichment in DFPCs was similar to that in PDLSCs, which is consistent with the scatter plots shown in Fig. 1a

The DNA methylation profiles of the genomes of the P2 DFPCs, PDLSCs, and DPSCs were investigated using Infinium HumanMethylation450. The standard index of DNA methylation at a specific CpG site is *β* (*β* = *M*/(*M* + *U* + 100)). *M* and *U* represent the methylated and unmethylated signal intensities, respectively. Three curves and the corresponding formulae for each comparison pair were obtained according to *β* value scatter plots of 480,000 DNA methylation sites in the three cell types (Fig. 1a). The correlation coefficient values (all *R*^2^ > 0.95) and curve fitting suggested that the DNA methylation profiles of DFPCs, PDLSC, and DPSCs were highly consistent. Moreover, the highest correlation coefficient value (*R*^2^ = 0.965) was observed between DFPCs and PDLSCs, indicating that these cell types had the highest similarity in DNA methylation profiles. Moreover, partial hierarchical clustering and a heat map of the 450K DNA methylation data shown in Fig. 1b revealed that DFPCs and PDLSCs have similar DNA methylation patterns, which is consistent with the scatter plots.

**Fig. 2:**
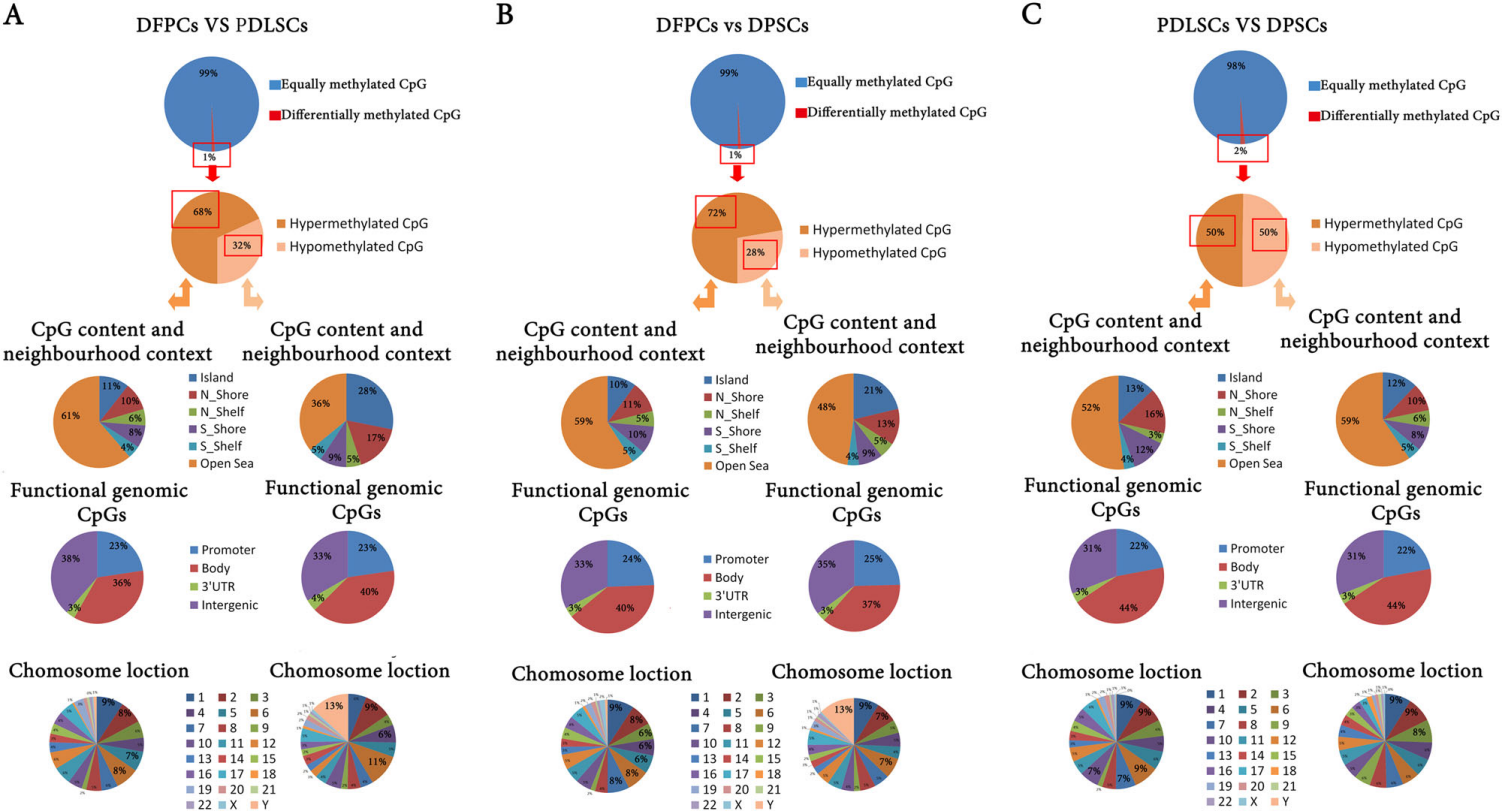
DNA methylation patterns in DFPCs, PDLSCs, and DPSCs. **a** Comparison of DFPCs and PDLSCs. **b** Comparison of DFPCs and DPSCs. **c** Comparison of PDLSCs and DPSCs. The percentages of differentially methylated CpG sites, distributions of hypermethylation and hypomethylation sites, and distributions of hypermethylated/hypomethylated CpGs in HCT-116 according to the CpG content and neighborhood context, functional genomic distribution, and chromosome location

The DNA methylation profiles were further compared among the three odontogenic stem cell lines. Of the 485,512 CpGs investigated, significant differences (*β* value >0.2, ratio >2 or <0.5) were observed at 1–2% of all sites (4758, 5448, and 7934 CpGs) between DFPCs and PDLSCs, DFPCs and DPSCs, and PDLSCs and DPSCs (Fig. 2). Most importantly, 68–72% (3244 and 3936 CpGs) of the DNA methylation sites were CpG hypermethylated sites in the DFPC/PDLSC and DFPC/DPSC comparisons, whereas hypomethylation only accounted for 28–32% (1512 and 1514 CpGs) (Fig. 2a, b). However, the hyper- and hypomethylation percentages in PDLSCs and DPSCs were identical (Fig. 2c). Regarding the type of CpG site, most hypermethylation sites occurred at a CpG island (10–14%; 344, 397, and 554 CpGs), in addition to an open sea area type. Regarding the location of the CpG sites, the hypermethylation sites were mainly located in the body of the gene (36–44%; 1157, 1558 and 1740 CpGs) in addition to the intergenic area. Moreover, DNA hypomethylation events also occurred mainly at CpG islands in addition to an open sea area at a range of 12–28% (426, 322, and 464 CpGs) and in the body of the gene (35–40%; 604, 558, and 1403 CpGs). Regarding the chromosomal location, the hypermethylation events were mainly located on chromosomes 1, 2, 5, 6, and 7 in each comparison of the three stem cell lines. In addition, the hypomethylation status mainly occurred on chromosome Y.

### Functional gene ontology classification analysis

**Fig. 3:**
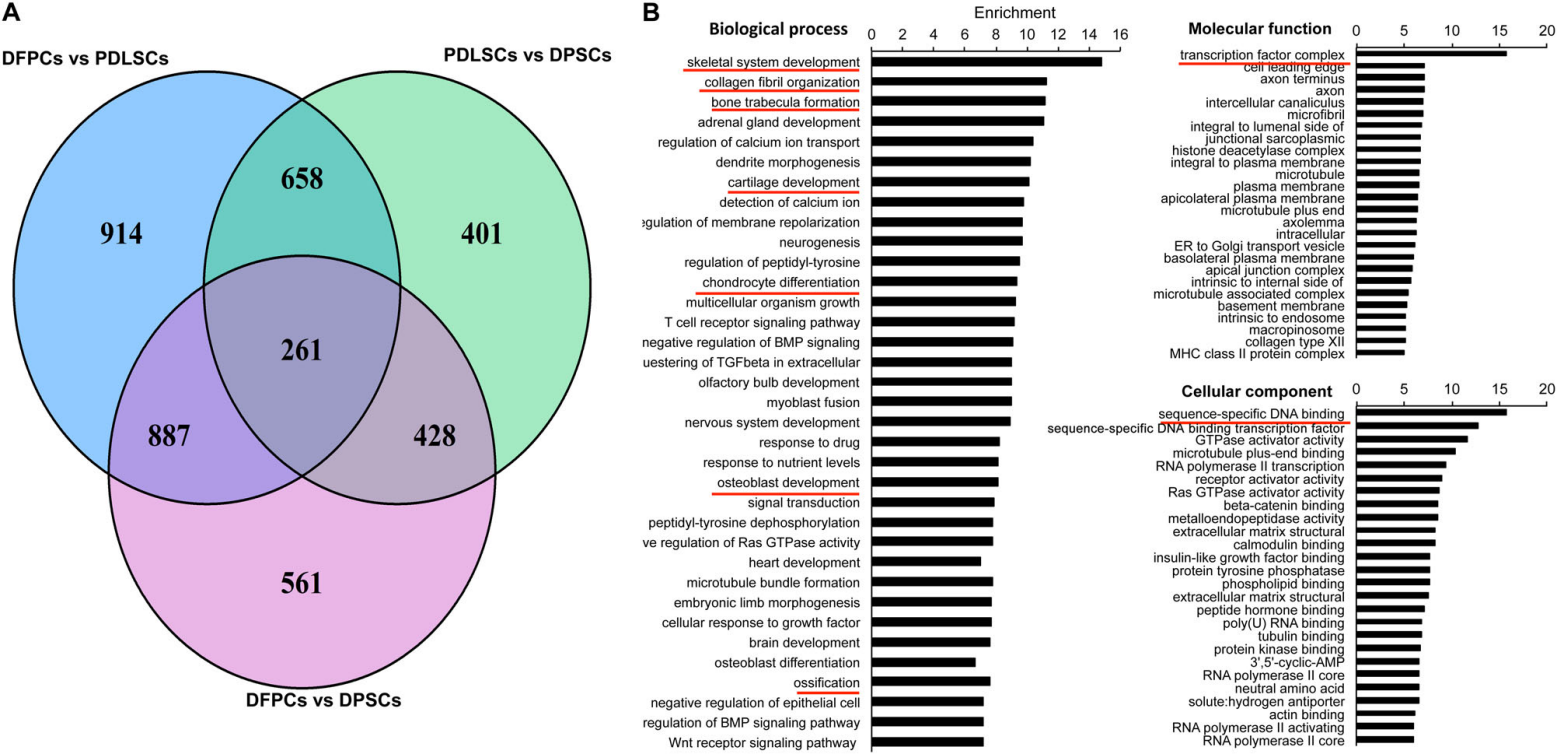
GO classification of genes with significantly different DNA methylation statuses among the cell lines. **a** Venn diagram of genes with significantly different methylation statuses between two groups. **b** Differential genes were related to many biological processes, including developmental-related processes, such as skeletal system, cartilage, nervous system, and osteoblast development. Genes with differentiation-related functions, such as fat cell and chondrocyte differentiation, also exhibited significant differences in methylation status. In addition, osteogenic-related biological processes, such as collagen fibril organization, bone trabecular formation, and detection of calcium ion processes, contained genes with significantly different methylation statuses

The methylated levels of many genes significantly differed. In general, the greatest number of genes differed between PDLSCs and DPSCs (Fig. 3a), which is consistent with the results shown in Fig. 1 and the differential methylation site distribution shown in Fig. 2. A GO analysis was then conducted using the results of the genomic DNA methylation analysis. The GO analysis classified the genes that showed a significantly different DNA methylation status and included many biological processes and functions (Fig. 3b). The comparison of DFPCs, PDLSCs, and DPSCs revealed that the differentially methylated genes were related to many biological processes, including development, such as skeletal system development, cartilage development, nervous system development, and osteoblast development. Differentiation-related functions, such as fat cell and chondrocyte differentiation, also exhibited differences. In addition, osteogenic-related biological processes, such as collagen fibril organization, bone trabecular formation, and detection of calcium ion processes, were included in the list.

### DNA methylation statuses of cell surface antigens, microRNAs, and transcription factors in osteogenic-related pathways differ

**Fig. 4:**
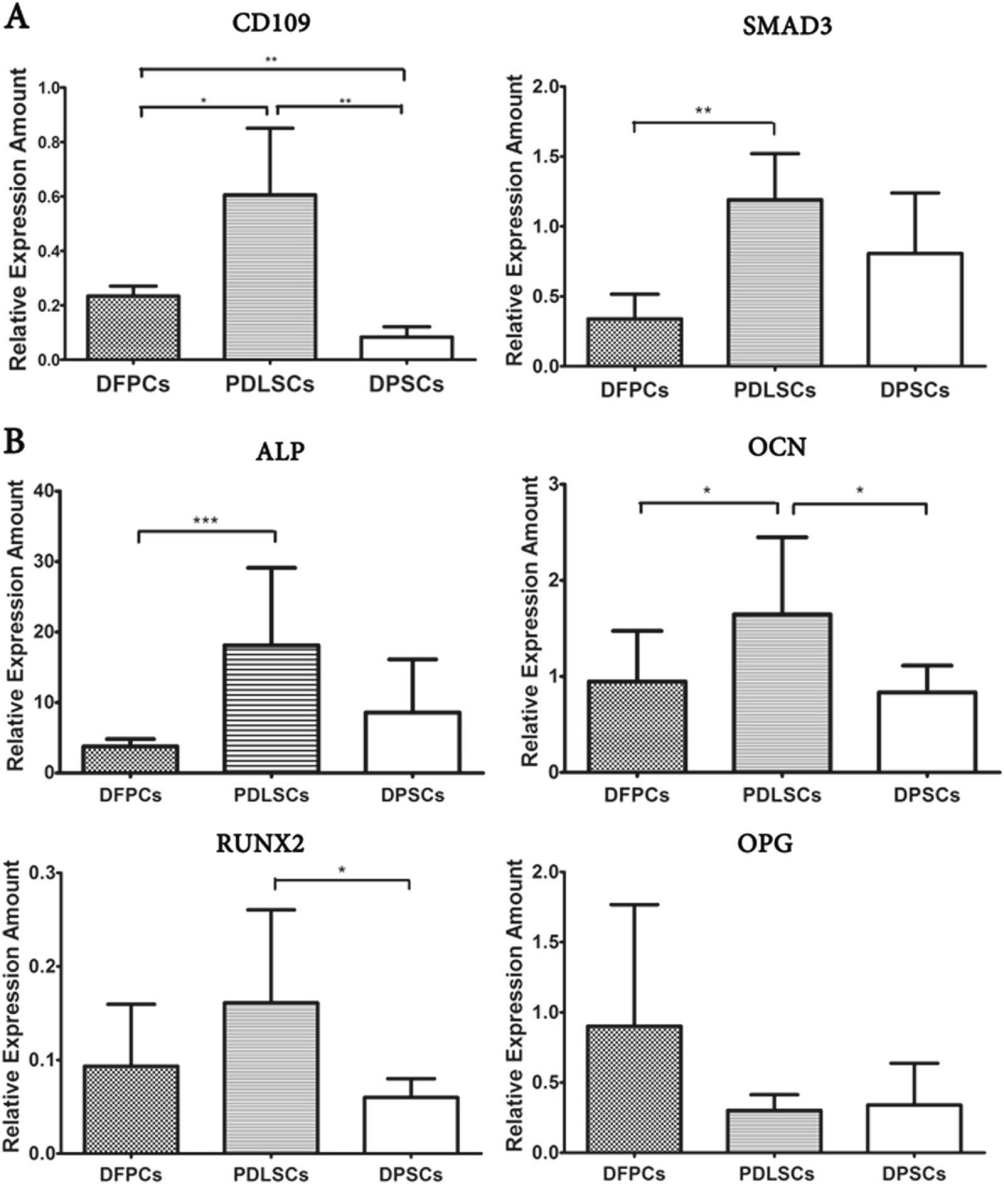
Expression of CD109, SMAD3, and other factors associated with osteogenic-related pathways as determined by RT-PCR. **a** CD109 and SMAD3 showed differential expression levels among the three groups; **b** Expression levels of ALP, OCN, and RUNX2 were considerably higher in PDLSCs than those in the other two cell lines (*p < 0.05, **p < 0.01)

Several cluster of differentiation (CD) surface antigens, which are important cell surface markers and microRNAs, which play a crucial role in the regulation of cell fate, exhibited differential DNA methylation statuses (Appendix Table 3). In this study, STRO-1 and CD146 were used as general surface markers of stem cells to identify the three cell types. In addition, the results presented in Appendix Figure 1A and B showed that all three stem cell types expressed STRO-1 and CD146, which is consistent with previous reports. In addition, these two surface antigens did not significantly differ (*p* > 0.05) among the three cell lines. Furthermore, CD109, which is a surface antigen, showed significant differences in the DNA methylation analysis in our study and is a gene associated with osteogenic function. Thus, we detected CD109 along with STRO-1 and CD146 using flow cytometry. Appendix Figure 1C presents the significant differences in the mean fluorescence intensity ratios (MFIR) of CD109 expression between DFPCs and PDLSCs (*p* = 0.017) and PDLSCs and DPSCs (*p* = 0.006), indicating that the osteogenic function of these three stem cells may differ. In addition, because SMAD3 is an important transcription factor that was among the genes with significantly different DNA methylation statuses, SMAD3 was evaluated at the transcriptional level along with CD109 using RT-PCR. The expression levels of CD109 and SMAD3 were significantly higher in PDLSCs than those in the other two cell lines, thus validating the DNA methylation profiles. Additionally, other factors in the osteogenic-related pathways that play vital roles in the regulation of bone formation were determined at the transcriptional level. The expression levels of ALP, OCN, and RUNX2 were considerably higher in PDLSCs than those in the other two cell lines (*p* < 0.05), which is consistent with the CD109 and SMAD3 data (Fig. 4).

### DPSCs, PDLSCs, and DFPCs exhibit different osteogenic capacities in vitro and in vivo

Several gene categories related to osteogenic function were revealed in the GO analysis, indicating that the osteogenic potential varied among the three cell lines. Thus, the bone formation capacities were investigated in vitro and in vivo. In vitro, the morphology and growth patterns of the three cell lines changed from a spindle-shaped fibroblast morphology to a stellate and irregular morphology following culture in osteo-inductive medium. In addition, all three cell populations formed extracellular mineralized matrices that were positively stained by Alizarin Red S following culture in osteogenic medium for 14 days. Each cell line also formed multiple individual clusters. The relative intensity of the staining in PDLSCs was significantly higher than that in DFPCs and DPSCs (*p* < 0.05) (Appendix Figure 2A). Mineralization was evaluated by ALP staining; the ALP activity was highest in PDLSCs, followed by DFPCs and DPSCs (Appendix Figure 2B).

**Fig. 5:**
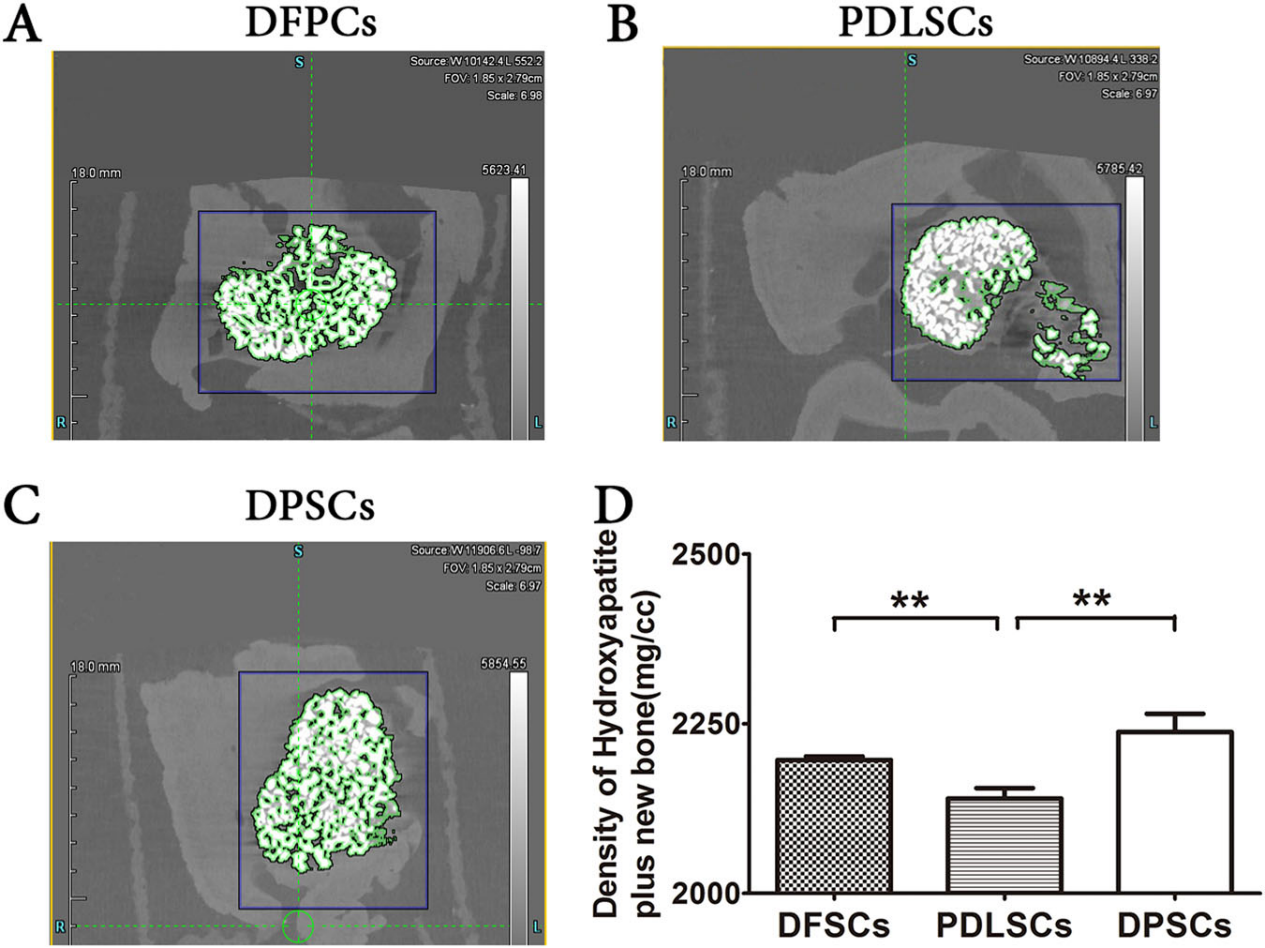
Micro-CT images of new bone formation in sediment from cultures of DFPCs, PDLSCs, and DPSCs mixed with hydroxyapatite 8 weeks after subcutaneous transplantation into the dorsal surface of immune-compromised male mice. **a** New bone formation by DFPCs. **b** New bone formation by PDLSCs. **c** New bone formation by DPSCs. The white substance shown in **a**, **b**, and **c** is hydroxyapatite, and the surrounding green areas are new bone. **d** The overall density of hydroxyapatite plus osteoid after the transplantation of DFPCs, PDLSCs, and DPSCs. The overall density of hydroxyapatite plus osteoid induced by the transplantation of PDLSCs was significantly lower than that in the other two cell lines (**p < 0.01). DPSCs and DFPCs did not significantly differ

To assess the bone formation capacity of the human DFPCs, PDLSCs, and DPSCs in vivo, cell sediments mixed with hydroxyapatite were transplanted subcutaneously into the dorsal surface of immune-compromised male mice. After 8 weeks, all samples were subjected to micro-CT scanning, which revealed considerable mineral formation. Additionally, PDLSCs showed a lower “density of hydroxyapatite plus osteoid” than the other two cell lines (*p* < 0.05). The osteoid formed by the stem cells grown along the hydroxyapatite after the cells were transplanted. Because the density of the hydroxyapatite is the greatest and the density of the osteoid is much lower than that of mature bone, the overall density will decrease if more osteoid is formed by the stem cells. Thus, PDLSCs that showed the lowest “density of hydroxyapatite plus osteoid” possessed the highest osteogenic potential. No significant difference in mineralization was observed between DFPCs and DPSCs (Fig. 5).

## Discussion

Human tooth development comprises several predetermined morphological stages involving sequential and reciprocal interactions between the epithelium and mesenchyme^20^. The mesenchyme originates from cranial neural crest cells that generate a complex of multipotent progenitors or stem cells^21,22^. These cells migrate and undergo diverse fates to generate structures with a unique morphology and function, such as enamel, dentin, periodontal ligaments, and dental pulp^23,24^. In addition, odontogenic stem cells play a critical role in tooth tissue repair and homeostasis throughout the life of the tooth^25^.

Due to these embryonic features and several other advantages, autologous odontogenic MSCs are of great interest in biological tissue engineering and regenerative medicine. These stem cell types are present in various tooth tissues, indicating that their properties may differ^26,27^. However, the differences among the three dental MSC types in terms of application in tissue engineering remain unclear.

DNA methylation is a vital epigenetic mechanism that is essential in early embryonic development and differentiation due to its role in the regulation of gene expression patterns. Thus, in this study, we aimed to investigate the influence of the DNA methylation profiles of these stem cells on their osteogenic potential, which is of great importance for bone and tissue regeneration. The HumanMethylation450 BeadChip was used in this study because it provides genome-wide coverage and enables a high-throughput low-cost analysis.

In addition to DNA methylation, histone modification is a critical regulatory component of epigenetic mechanisms affecting the fate of stem cells^28,29^. Histone modification occurs through the activity of specific nuclear enzymes, including the histone H3 lysine 4 tri-methylation (H3K4me3) and H3K27 acetylation (H3K27ac) initiation of gene activation, H3K9me3 and H3K27me3 initiation of gene repression, etc.^30^ Studies have showed that these histone modifications are crucial for MSC osteogenic differentiation^31^. In addition, another study suggested that the posttranscriptional modification of histone by acetylation plays an important role in the osteogenic differentiation of PDLSCs^32^. Histone acetyltransferase GCN5 has been shown to regulate the osteogenesis of PDLSCs^33^. Furthermore, the acetylation of H3K9 and H3K14 could promote osteogenic differentiation in PDLSCs^34^.

To date, numerous studies have focused on odontogenic stem cells. However, these studies have explored different aspects. For example, in the study conducted by Eleuterio^35^, the authors compared the proteomic profiles of in vivo cultured human PDLSCs, DPSCs, and BMSCs to define the molecular features of odontogenic stem cells. Their results exhibited qualitative similarities in the proteome profiles among the three stem cells. In addition, the authors concluded that oral stem cells could have a different cell lineage potency than BMSCs, which may explain their ability of neuronal differentiation, which is consistent with their neural crest origin. However, the authors did not perform relevant functional experiments for further verification. Another study^8^ performed by Guo et al. compared the differentiation potential of dental follicle cells (DFCs) and dental papilla cells (DPCs) and indicated that the DPSCs were better at vascular differentiation and that DFPCs possessed a stronger osteogenic differentiation potential according to the analysis of the differential protein expression. In contrast with the two above-mentioned studies, we mainly analyzed the differential DNA methylation of DFPCs, PDLSCs, and DPSCs and observed evidence of their differential osteogenic abilities. Our study performed a DNA methylation analysis and then traced the osteogenic-related activities of three different odontogenic stem cell types, which differs from most previous studies.

Overall, the general similarity and specific differences in the DNA methylation profiles of the three odontogenic cell lines likely indicated that the distinct developmental stages or microenvironment might be related to the DNA methylation patterns. For instance, the DPSCs are capable of differentiating into multilineage cells, including neuron-like cells, whereas PDLSCs are highly likely to differentiate into cementoblasts and osteoblasts^19,36^.

The GO classification analysis showed that various biological processes significantly differed among the three cell lines, including developmental-, differentiation-, and osteogenic-related biological processes. This finding suggests that these cell types play different roles during development and differentiation. In addition, the differences in the osteogenic capacity were demonstrated in the in vitro and in vivo experiments. Thus, the similar gene methylation profiles between DFPCs and PDLSCs and greater osteogenic capacity of PDLSCs may assist clinicians or researchers in making more appropriate decisions in clinical trials or bone regeneration research studies. The characteristics of differentiation will be explored in our future studies.

Notably, although the dental follicles were obtained from developing teeth at a relatively early developmental stage, their osteogenic potential was not maximal. Therefore, the early developmental stages may not be directly related to the osteogenic capacity because PDLSCs, but not DFPCs, demonstrated a greater osteogenic capability. Furthermore, the identification of dental MSCs and the factors involved in the determination of their fate is a considerable accomplishment^37^. Odontogenic stem cells are promising biomaterials for tissue engineering and regenerative medicine; their potential applications should be further explored.

## Conclusions

In this study, the DNA methylation profiles and distribution of three cell lines were found to be similar, and the highest similarity was observed between DFPCs and PDLSCs. However, specific differences were identified among the cell lines investigated. Specifically, various cell surface antigens and microRNAs significantly differed, and GO analysis revealed that several gene categories with significantly different DNA methylation statuses were related to osteogenic functions, suggesting that the osteogenic potential varies among the three cell lines. Moreover, compared with DFPCs and DPSCs, PDLSCs exhibited higher transcriptional levels of osteogenic-related factors, a higher osteogenic potential in vitro and an enhanced new bone formation capacity in vivo (*p* < 0.05). These results suggest that the differential DNA methylation profiles may indicate the osteogenic potential of these three odontogenic cell populations.

## Electronic supplementary material

Appendix
