# Supplementary material for: DNA methylation profile is associated with the osteogenic potential of three distinct human odontogenic stem cells
Source: Signal Transduct Target Ther. 2018 Jan 12;3:1. doi: 10.1038/s41392-017-0001-6 (PMC5837092; doi:10.1038/s41392-017-0001-6)
Supplement: Supplementary file 1 — Appendix [file 41392_2017_1_MOESM1_ESM.pdf]

Appendix Table 1. Detail information of the individual sample

Cell type	Age	Sex	Site
DFSCs1	22	Male	Third molar
DFSCs2	16	Female	Third molar
DFSCs3	19	Female	Third molar
DFSCs4	17	Male	Third molar
PDLSCs1	19	Female	Premolar
PDLSCs2	14	Male	Premolar
PDLSCs3	12	Female	Premolar
PDLSCs4	15	Male	Premolar
DPSCs1	13	Male	Premolar
DPSCs2	19	Female	Premolar
DPSCs3	25	Female	Premolar
DPSCs4	16	Male	Premolar

DFPCs, dental follicle progenitor cells; PDLSCs, periodontal ligament stem cells; DPSCs, dental pulp stem cells;

Appendix Table 2. Primers for Real-time PCR

Human genes name	Forward primer sequence (5'→3')	Reverse primer sequence (5'→3')
GAPDH[1]	CGACAGTCAGCCGCATCTT	CCAATACGACCAAATCCGTTG
CD109[2]	TGTCTCCTTCCCACATCCTC	CAGCTTCTTTCCCAAACTGC
SMAD3[3]	GCGTGCGGCTCTACTACATC	GCACATTCGGGTCAACTGGTA
COL1α2[4]	AAGGTCATGCTGGTCTTGCT	GACCCTGTTCACCTTTTCCA
ALP[5]	AGCACTCCCACTTCATCTGGAA	GAGACCCAATAGGTAGTCCACATTG
OCN[6]	CCTGAAAGCCGATGTGGT	AGGGCAGCGAGGTAGTGA
RUNX2[7]	AGGAATGCGCCCTAAATCACT	ACCCAGAAGGCACAGACAGAAG
OPG[8]	GGAACCCCAGAGCGAAATACA	CCTGAAGAATGCCTCCTCACA

Referrences

1. Xiao E, Li JM, Yan YB, An JG, Duan DH, Gan YH, et al. Decreased osteogenesis in stromal cells from radiolucent zone of human TMJ ankylosis. J Dent Res. 2013 May; 92(5):450-455.
2. Li J, Xin J, Zhang L, Wu J, Jiang L, Zhou Q, et al. Human hepatic progenitor cells express hematopoietic cell markers CD45 and CD109. Int J Med Sci. 2013 Dec 21;11(1):65-79.
3. Xiao E, Li JM, Yan YB, An JG, Duan DH, Gan YH, et al. Decreased osteogenesis in stromal cells from radiolucent zone of human TMJ ankylosis. J Dent Res. 2013 May; 92(5):450-455.
4. http://medgen.ugent.be/rtprimerdb/assay_report.php?assay_id=268.
5. S Zhou, J Zhang, H Zheng, Y Zhou, F Chen, J Lin. Inhibition of mechanical stress-induced NF-jB promotes bone formation. Oral diseases 2013,19,59-64.
6. Xiao E, Li JM, Yan YB, An JG, Duan DH, Gan YH, et al. Decreased osteogenesis in stromal cells from radiolucent zone of human TMJ ankylosis. J Dent Res. 2013 May; 92(5):450-455.
7. S Zhou, J Zhang, H Zheng, Y Zhou, F Chen , J Lin. Inhibition of mechanical stress-induced NF-jB promotes bone formation. Oral diseases 2013,19,59-64.
8. Xiao E, Li JM, Yan YB, An JG, Duan DH, Gan YH, et al. Decreased osteogenesis in stromal cells from radiolucent zone of human TMJ ankylosis. J Dent Res. 2013 May; 92(5):450-455.

Appendix Table 3. Differentially expressed cell surface antigens and microRNA

A. Differentially expressed cell surface antigens

Gene name	TargetID	DFPCs	PDLSCs	DPSCs	Gene group
CD1D	cg05046020	0.36185	0.11847	0.75314	TSS1500
CD9	cg19615684	0.67753	0.25098	0.77561	Body
CD14	cg19008097	0.45304	0.21177	0.47014	1stExon
CD24	cg05230942	0.24279	0.53177	0.61252	TSS1500
CD40	cg01943874	0.17583	0.19259	0.40161	1stExon
CD44[1]	cg02663352	0.54902	0.08552	0.50893	Body
CD59	cg09864245	0.35489	0.09473	0.4703	5'UTR
CD96	cg23670794	0.59176	0.61224	0.21088	Body
CD109[2]	cg23004174	0.70565	0.16685	0.79166	TSS1500
CD200	cg13910460	0.43825	0.21434	0.50284	Body
CD247	cg09179987	0.66452	0.66961	0.33074	Body

B. Differentially expressed microRNA

Gene Name	TargetID	DFPCs	PDLSCs	DPSCs	Gene Group
MIR99A	cg21750426	0.12248	0.63495	0.59915	Body
MIR128-2	cg21384588	0.7782	0.24382	0.70616	TSS200
MIR128-2	cg23220346	0.66345	0.11689	0.55025	Body
MIR128-2	cg24102420	0.65101	0.20691	0.57857	TSS200
MIR193B[3]	cg03295417	0.6635	0.31098	0.74205	Body
MIR200C[4]	cg16642299	0.42643	0.20744	0.12538	TSS1500
MIR346[5]	cg14368220	0.10148	0.70067	0.67317	TSS200
MIR548H3	cg22902499	0.34547	0.44027	0.103	Body
MIR559	cg15792957	0.7082	0.68858	0.28084	TSS1500
MIR563	cg06368401	0.41599	0.5189	0.17445	TSS1500
MIR623	cg16193278	0.36819	0.11853	0.36398	Body
MIR874	cg04986004	0.13064	0.10859	0.35708	TSS1500
MIR1208	cg07018107	0.38719	0.11306	0.04765	Body
MIR1284	cg18558767	0.52006	0.17676	0.62371	TSS1500
MIRLET7C	cg19173502	0.18397	0.61023	0.53752	TSS1500

C. Other differentially expressed genes

Gene Name	TargetID	DFPCs	PDLSCs	DPSCs	Gene Group
MIR99A	cg21750426	0.12248	0.63495	0.59915	Body
MIR128-2	cg21384588	0.7782	0.24382	0.70616	TSS200
MIR128-2	cg23220346	0.66345	0.11689	0.55025	Body
MIR128-2	cg24102420	0.65101	0.20691	0.57857	TSS200
MIR193B[3]	cg03295417	0.6635	0.31098	0.74205	Body
MIR200C[4]	cg16642299	0.42643	0.20744	0.12538	TSS1500
MIR346[5]	cg14368220	0.10148	0.70067	0.67317	TSS200
MIR548H3	cg22902499	0.34547	0.44027	0.103	Body

MIR559	cg15792957	0.7082	0.68858	0.28084	TSS1500
MIR563	cg06368401	0.41599	0.5189	0.17445	TSS1500
MIR623	cg16193278	0.36819	0.11853	0.36398	Body
MIR874	cg04986004	0.13064	0.10859	0.35708	TSS1500
MIR1208	cg07018107	0.38719	0.11306	0.04765	Body
MIR1284	cg18558767	0.52006	0.17676	0.62371	TSS1500
MIRLET7C	cg19173502	0.18397	0.61023	0.53752	TSS1500

Referrences

1. Yeh Y, Yang Y, Yuan K. Importance of CD44 in the proliferation and mineralization of periodontal ligament cells. J Periodontal Res 2014 Feb 12.
2. Wang Y, Inger M, Jiang H, Tenenbaum H, Glogauer M. CD109 plays a role in osteoclastogenesis. PLoS One 2013;8(4):e61213.
3. Ukai T, Sato M, Akutsu H, Umezawa A, Mochida J. MicroRNA-199a-3p, microRNA-193b, and microRNA-320c are correlated to aging and regulate human cartilage metabolism. J Orthop Res 2012 Dec;30(12):1915-1922.
4. Wendlandt E, Graff J, Gioannini T, McCaffrey A, Wilson M. The role of microRNAs miR-200b and miR-200c in TLR4 signaling and NF-κB activation. Innate Immun 2012 Dec;18(6):846-855.
5. Wang Q, Cai J, Cai X, Chen L. miR-346 regulates osteogenic differentiation of human bone marrow-derived mesenchymal stem cells by targeting the Wnt/β-catenin pathway. PLoS One 2013;8(9):e72266.

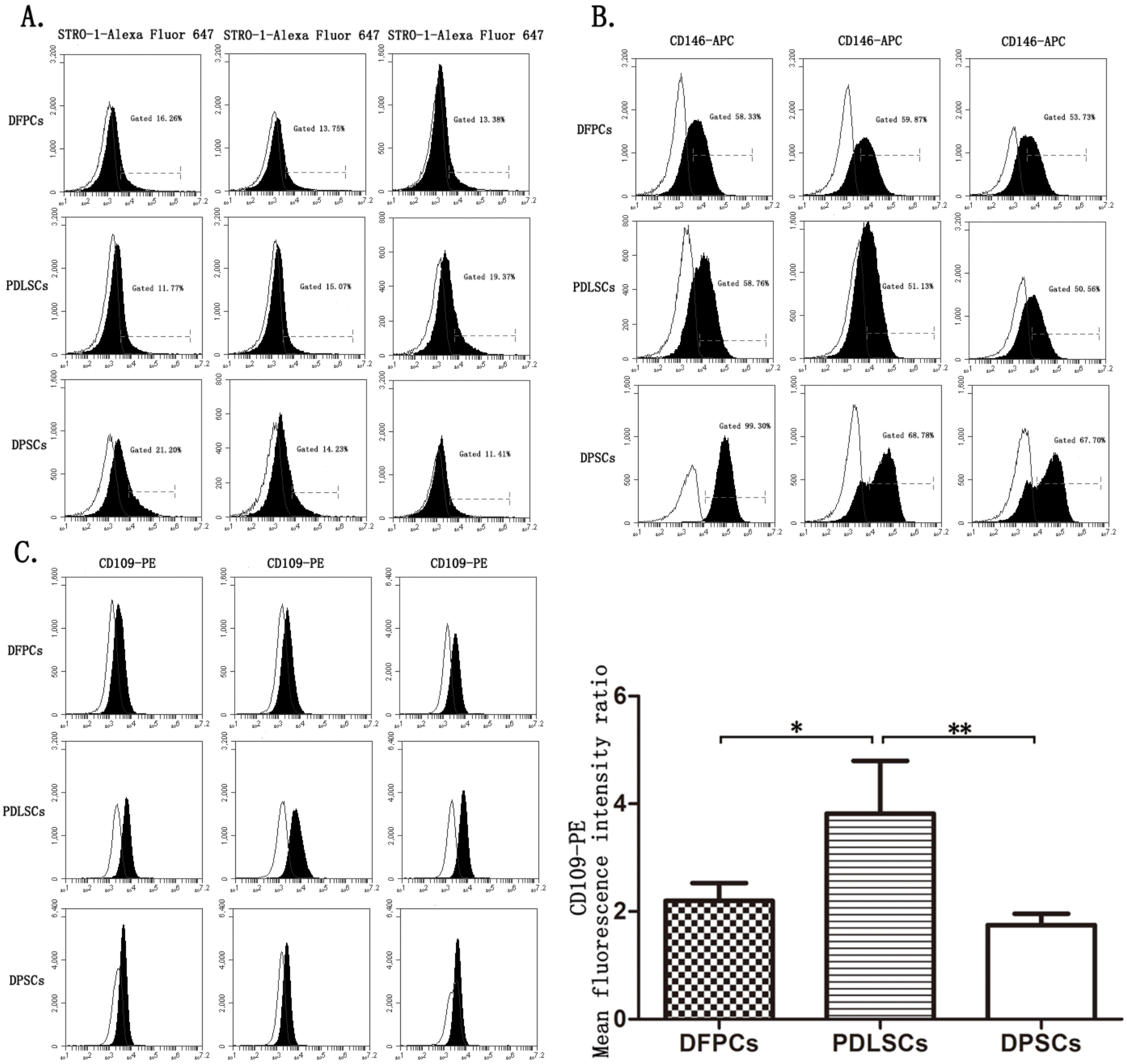

Appendix Figure 1. Surface antigen expression in DFPCs, PDLSCs and DPSCs as determined by flow cytometry. A. DFPCs, PDLSCs and DPSCs stained positive for the MSC marker STRO-1. B. CD146 expression in DFPCs, PDLSCs and DPSCs. C. CD109 expression and mean fluorescence intensity ratios (MFIR) in DFPCs, PDLSCs and DPSCs (*$p < 0.05$, **$p < 0.01$).

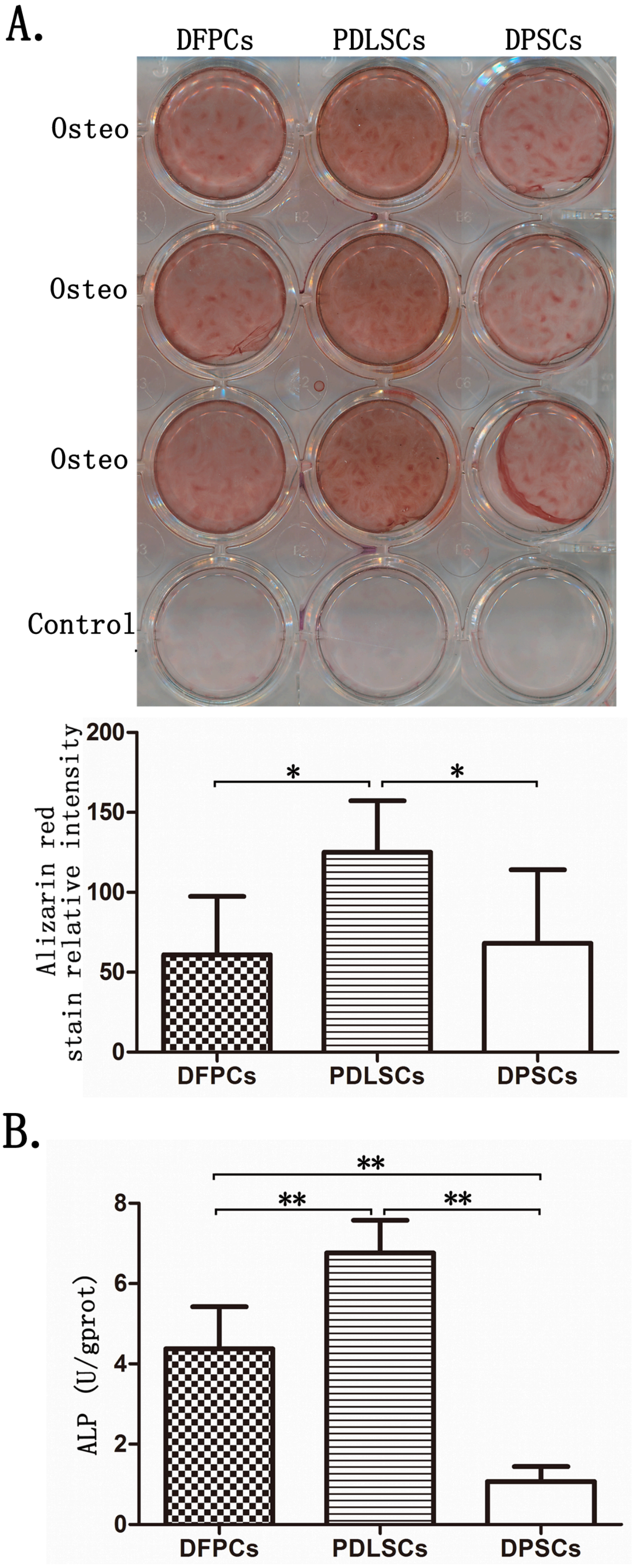

Appendix Figure 2. Formation of mineralised nodules and ALP activities in DFPCs, PDLSCs and DPSCs. A. Quantitative comparison of Alizarin red staining in DFPCs, PDLSCs and DPSCs after 2 weeks of culture in osteogenic medium (*p < 0.05, **p < 0.01). B. ALP activity in DFPCs, PDLSCs and DPSCs at day 7 of culture in osteo-inductive medium (*p<0.05, **p<0.01).
